# Identification of large disjoint motifs in biological networks

**DOI:** 10.1186/s12859-016-1271-7

**Published:** 2016-10-06

**Authors:** Rasha Elhesha, Tamer Kahveci

**Affiliations:** CISE Department, University of Florida, 432 Newell Dr, Gainesville, Florida, 32611 USA

**Keywords:** Biological networks, Motif discovery, Overlap graph, Subgraph isomorphism

## Abstract

**Background:**

Biological networks provide great potential to understand how cells function. Network motifs, frequent topological patterns, are key structures through which biological networks operate. Finding motifs in biological networks remains to be computationally challenging task as the size of the motif and the underlying network grow. Often, different copies of a given motif topology in a network share nodes or edges. Counting such overlapping copies introduces significant problems in motif identification.

**Results:**

In this paper, we develop a scalable algorithm for finding network motifs. Unlike most of the existing studies, our algorithm counts independent copies of each motif topology. We introduce a set of small patterns and prove that we can construct any larger pattern by joining those patterns iteratively. By iteratively joining already identified motifs with those patterns, our algorithm avoids (i) constructing topologies which do not exist in the target network (ii) repeatedly counting the frequency of the motifs generated in subsequent iterations. Our experiments on real and synthetic networks demonstrate that our method is significantly faster and more accurate than the existing methods including SUBDUE and FSG.

**Conclusions:**

We conclude that our method for finding network motifs is scalable and computationally feasible for large motif sizes and a broad range of networks with different sizes and densities. We proved that any motif with four or more edges can be constructed as a join of the small patterns.

**Electronic supplementary material:**

The online version of this article (doi:10.1186/s12859-016-1271-7) contains supplementary material, which is available to authorized users.

## Introduction

Biological networks describe how molecules interact to carry out various cellular functions. One common way to represent these networks is to use graphs, where the nodes and the edges represent the interacting molecules and the interactions between these molecules respectively [1]. Studying biological networks has great potential to help understand how cells function and how they respond to extra-cellular stimulants. Such studies have already been used successfully in many applications. Characterizing the variations in drug resistance of different cell lines [2], or identifying the pathways serving similar functions across different organisms [3, 4] are only few examples among many.

Motifs are frequent topological patterns in a given network [5]. Identifying motifs has been one of the key steps in understanding the functions served by biological networks such as gene regulatory or protein interaction networks [6–8]. Motifs can be used to uncover the basic structure and design principles of a network [9]. They are also often considered as the basic building blocks of a network [5] and one of the network local properties [10]. Thus, they can be used to classify networks [11] into functional sub-units. It is worth noting that motifs have been used in various applications like prediction of regulatory elements in genomic sequences [12].

Despite the fact that studying motifs is of utmost importance for network analysis, motifs identification remains to be a computationally hard problem [13]. The roots of the challenges behind motif discovery arise from several reasons. First, even when the motif topology is given, counting motif frequency (i.e. the number of occurrences of this motif), requires solving the subgraph isomorphism problem, which is NP-Complete [14]. Furthermore, when the motif topology is not known in advance, trying out all alternative topologies is infeasible as the number of such topologies increases exponentially with the number of edges in the motif.

There are two ways for motif frequency formulation; (i) allow for different copies of the same motif to overlap (i.e., share nodes or edges) or (ii) count disjoint copies of the motif under consideration. Most of the existing methods in the literature on motif counting follow the first formulation. This formulation however has a fundamental drawback arising from the fact that it does not have *downward closure* property. Briefly, this means that the motif frequency does not decrease monotonically as the motif size increases. We discuss this drawback in detail in Sections “Summary of existing methods” along with why it makes it impossible to determine the largest sized motif in a given network. Several algorithms use the second formulation to compute the frequency of a given motif (e.g., [15]). Those algorithms, however, do not scale to large networks. Also, they are limited to small motifs as their time complexities grow exponentially with motif size. We elaborate on these methods in Section “Summary of existing methods” as well.

In this paper, we address the problem of finding motifs in a given network. More specifically, given a target network and a motif size (i.e., number of nodes in the motif), we aim to find the motifs of that size which have a frequency above a user specified threshold in that target network. Unlike most of the methods in the literature, we use the second formulation of motif counting described above, where no two copies of the same motif share an edge, to compute the frequency.

We develop a novel and scalable algorithm to solve the motif identification problem. The central idea of our method, which stands out among the existing literature, is to use a small set of patterns, called the *basic building patterns*. We prove that any motif with four or more edges can be constructed as a combination of these patterns. Following from this observation, our method first finds instances of these patterns. It then iteratively grows motifs by joining known motifs at that iteration with the instances of these patterns. Our algorithm develops efficient mechanisms to avoid a significant fraction of the costly isomorphism tests while growing new motifs. Counting non-overlapping instances of a given motif is a computationally challenging task that requires solving maximum independent set (MIS) problem which is known to be NP-complete [13]. We introduce a new and efficient strategy for this purpose. This strategy avoids enumerating the overlapping motif instances. It does this by algebraically computing the overlap count based on the neighbors of the motif nodes in the target network. Our experiments on both protein-protein interaction (PPI) and synthetic networks demonstrate that our method is significantly faster and more accurate than the existing methods. In addition, the increase in the running time of our algorithm is dramatically less than that of the competing methods as the motif size grows.

The rest of this paper is organized as follows. We present the key definitions needed to discuss our method and the related literature in Section “Background”. We describe our motif discovery algorithm in Section “Methods”. We experimentally evaluate our method and compare it to the existing algorithms in Section “Results and discussion”. We end with a brief conclusion in Section “Conclusions”.

## Background

In this section, we provide the definitions and the terminology needed to describe our method (Section “Definitions and notation”). We then summarize the key literature tackling similar problems to the one considered in this paper (Section “Summary of existing methods”).

### Definitions and notation

**Fig. 1:**
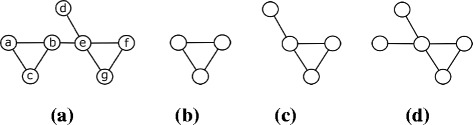
**a** A graph *G* that contain seven nodes {a, b, c, d, e, f, g} and eight edges {(a,b), (a,c), (b,c), (b,e), (e,d), (e,f), (f,g), (e,g)}. **b** A pattern with two embeddings in *G*, {(a,b), (a,c), (b,c)} and {(e,f), (f,g), (e,g)}. **c** A pattern with three embeddings in *G*, {(a,b), (a,c), (b,c), (b,e)}, {(e,f), (f,g), (e,g), (e,d)}, and {(e,f), (f,g), (e,g), (b,e)}. **d** A pattern that has one copy in *G*, {(b,e), (e,d), (e,f), (f,g), (e,g)}

We represent a given biological network using a graph denoted with *G*=(*V, E*). Here, the set of nodes *V* denotes the set of interacting molecules, and the set of edges *E* denotes the interactions among them. In the rest of this paper, we use the term graph to denote a biological network. Here, we focus on undirected graphs. Figure 1 a represents a graph that contains seven nodes and eight edges.

We say that a graph is *connected* if there is a path between all pairs of its nodes. We say that a graph *S*=(*V*_*S*_,*E*_*S*_) is a *subgraph* of *G* if *V*_*S*_⊆*V* and *E*_*S*_⊆*E*. In the rest of this paper, we only consider connected subgraphs. Thus, to simplify our terminology, we use the term subgraph instead of connected subgraph. Notice that a subgraph of a given graph can be uniquely determined by the set of edges *E*_*S*_ of that subgraph as all of its nodes are connected.

We say that two subgraphs $\phantom {\dot {i}\!}S_{1} = (V_{S_{1}}, E_{S_{1}})$ and $\phantom {\dot {i}\!}S_{2} = (V_{S_{2}}, E_{S_{2}})$ of *G* are *identical* if they have the same set of edges. A less constrained association between two subgraphs is *isomorphism*. Two subgraphs *S*_1_ and *S*_2_ are isomorphic if the following condition holds: There exists a bijection $f: V_{S_{1}} \rightarrow V_{S_{2}}$ such that $\forall ({u,v}) \in E_{S_{1}}, \iff (f(u), f(v)) \in E_{S_{2}}$.

We say that two subgraphs *S*_1_ and *S*_2_ *overlap* if they share at least one edge (i.e., $E_{S_{1}} \cap E_{S_{2}} \neq \emptyset $). In Fig. 1 a, consider the four subgraphs *S*_1_, *S*_2_, *S*_3_, and *S*_4_ defined by the set of edges {(a,b), (a,c), (b,c), (b,e)}, {(e,f), (f,g), (e,g), (e,d)}, {(e,f), (f,g), (e,g), (b,e)}, and {(b,e), (d,e), (e,f), (e,g)} respectively. *S*_1_ and *S*_2_ are disjoint as they do not share any edges. *S*_1_ and *S*_3_ overlap as they share the edge (b,e). Similarly *S*_2_ and *S*_3_ overlap. All three subgraphs *S*_1_, *S*_2_, and *S*_3_ are isomorphic as they have the same topology. *S*_1_ and *S*_4_ are non-isomorphic as they do not satisfy the bijection function defined above.

Notice that isomorphism is a transitive relation. Thus, for a given subgraph *S* of *G*, the set of all subgraphs of *G* which are isomorphic to *S* defines an equivalence class. We represent the subgraphs in each equivalence class with a graph isomorphic to those in that equivalence class and call it a *pattern*. Figure 1 c shows the pattern that represents the equivalence class { *S*_1_, *S*_2_, *S*_3_}.

There are alternative definitions of the frequency of a pattern in a given graph. The classical frequency definition is the number of all subgraphs of the target graph which are isomorphic to the given pattern. This definition, also known as the *F*1 measure [16], counts all the subgraphs regardless of whether they overlap with each other or not. There are two other frequency definitions which avoid overlaps between different subgraphs. *F*2 measure counts the largest subset of subgraphs in a given equivalence class which do not share any edges with the rest of the subgraphs in that subset. It however allows them to share nodes. *F*3 measure is more stringent as it requires that no two subgraphs can share a node. Consider the pattern in Fig. 1 c and the target graph in Fig. 1 a. The frequency of this pattern in the target graph according to the *F*1 measure is three as it has three embeddings ({ *S*_1_, *S*_2_, *S*_3_}). On the other hand *F*2 is two { *S*_1_, *S*_2_}, and *F*3 is one (*S*_1_ or *S*_2_ or *S*_3_). From here on, we denote the *F*1, *F*2, and *F*3 counts of a motif *M* in graph *G* using the notations *F*1_*G*_(*M*), *F*2_*G*_(*M*), and *F*3_*G*_(*M*) respectively.

The *downward closure* property states that the frequency of a pattern should monotonically decrease as this pattern grows (by inserting new nodes or edges to it). More specifically, consider a function *f*() that operates on a pattern and returns a real number. Let us denote two patterns with *P*_1_ and *P*_2_. We say that the function *f*() has downward closure property if and only if *f*(*P*_2_)≤*f*(*P*_1_) for all (*P*_1_, *P*_2_) pairs where *P*_1_ is a subgraph of *P*_2_.

Under the light of these definitions, next we show that *F*1 measure is not downward closed. Consider the pattern *P*_1_ in Fig. 1 b. The frequency of *P*_1_ is two in the target graph in Fig. 1 a. Now consider the pattern *P*_2_ in Fig. 1 c which contains *P*_1_. Although *P*_1_ is a subgraph of *P*_2_, the frequency of *P*_2_ is three in the same graph (i.e., more than that of *P*_1_). Next, consider the pattern *P*_3_ in Fig. 1 d. *P*_3_ contains *P*_2_, and its frequency is only one (i.e., less than that of *P*_2_). This example demonstrates that the *F*1 measure not only fails to monotonically decrease, but it also fluctuates (i.e., its value may go up or down) as we grow the pattern (see [17, 18] for further discussions on this issue).

Unlike the *F*1 measure, *F*2 is downward closed. In the following, we formally prove this.

#### **Theorem 1.1**

Assume that we are given a graph *G*. Given two patterns *M* and $\bar {M}$ where *M* $\subset \bar {M}$, we have $F2_{G}(M) \geq F2_{G}(\bar {M})$.

#### *Proof*

To prove this, we consider the placement of each embedding of $\bar {M}$ in *G* according to *F*2 measure (i.e. non-overlapping embeddings). Notice that each embedding of $\bar {M}$ contains *M* as *M* $\subset \bar {M}$. From each of these embeddings, we remove the edges that are in $\bar {M}-M$. This leads to one embedding of *M* for each embedding of $\bar {M}$. Thus, the number of non-overlapping embeddings of *M* in *G* is at least as much as that of $\bar {M}$ in *G*. Therefore, $F2_{G}(M) \geq F2_{G}(\bar {M})$. □

Similarly, we say that *F*3 measure which also counts non-overlapping embeddings, is also downward closed.

Failure to satisfy the downward closure property has major implications on the correctness of motif identification. Traditional motif identification algorithms often grow a motif starting from an initial motif of a small number of edges (see Section “Summary of existing methods”). Should they employ the *F*1 measure, these algorithms cannot have an early stopping criteria as they grow motifs. This is because the frequency can go up as we grow motif even when the current motif frequency is low. Next, we formally define the problem considered in this paper.

**Problem definition** Given an input graph *G*=(*V, E*), the number of nodes in the target motif *μ*, and frequency threshold *α*, we aim to find all patterns of *μ* nodes which have frequency at least *α* in *G* under the frequency measure *F*2. The method we develop in this paper can however be easily extended to *F*3 as well (see Section “Finding MIS: Going from F1 to F2”).

### Summary of existing methods

We classify the literature on motif identification and counting, based on the underlying frequency measure. This is because the frequency measure dramatically changes the cost of counting motifs as well as how we can interpret the frequency of the underlying pattern. Most of the existing studies use *F*1 frequency measure to count the embeddings of a pattern in a given graph (e.g., [19–24]). These methods carry the drawbacks inherent in the *F*1 measure. First, *F*1 ignores the fact that different copies of the same motif can overlap due to the nodes and the edges they share. This can lead to artificially massive number of motif embeddings as the same node or edge can participate in multiple embeddings. To understand this better, consider the pattern and the graph in Figs. 1 c and 1 a respectively. *F*1 counts three copies of the pattern (*S*_1_, *S*_2_, and *S*_3_). Different nodes and edges however contribute to this count at different numbers. The edge (a, b) appears only in *S*_1_ while (b, e) appears in both *S*_1_ and *S*_3_.

Second and more importantly, the *F*1 measure is not downward closed. This is because as we grow a pattern by including new edges or nodes, its count as computed by *F*1 is not monotonic; it may decrease, stay the same, or increase. Lack of downward closure property makes it nearly impossible to decide if the motif found is the largest one in size while growing a pattern. Thus, using *F*2 is essential for the tractability of identifying frequent patterns. We use the *F*2 measure in this paper. Thus, the studies limited to the *F*1 measure are out of the scope of this paper.

Several algorithms tackle the problem of finding frequent patterns in multiple graphs. FSG [25] is one of the key methods in this class. These methods, however, do not count the number of occurrences of a pattern in each graph. They rather check if the given pattern appears at least once in each graph. Vanetik et al. [17] also addressed the same problem.

Finding frequent patterns or counting them without overlaps (i.e., using *F*2 or *F*3 measures) have received little attention in the literature. One of the existing algorithms in this category is SUBDUE [15]. Flexible Pattern Finder Algorithm (FPF) [16] detects frequent patterns using both *F*2 and *F*3. Two algorithms were proposed by Kuramochi and Karypis [26], named hSiGraM, vSiGraM. However, these algorithms are computationally expensive and do not scale to large graphs or motifs. We evaluate SUBDUE and FSG experimentally in Section “Results and discussion”.

## Methods

In this section we describe our method. Section “Algorithm overview” presents an overview of our algorithm. Section “Joining patterns to find larger patterns” explains the mechanism we use to grow motifs by joining smaller motifs. Section “Finding MIS: Going from F1 to F2” describes how we count disjoint motif instances. Section “Accelerating our algorithm through efficient filters” presents filtering techniques we implement to avoid costly isomorphism tests. Section “Complexity analysis” discusses the complexity analysis of our method.

### Algorithm overview

**Fig. 2:**
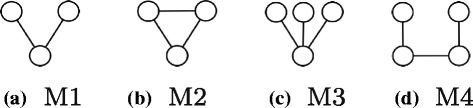
The four basic patterns used by our algorithm which represent all patterns of two (**a**) or three undirected edges (**b**, **c**, and **d**)

In this section, we provide an overview of our method for discovering motifs. At the heart of our method lie four unique graph patterns. We call them the *basic building patterns* for we use them as guide to construct larger motifs of arbitrary sizes and topologies. Figure 2 presents these basic building patterns. We explain why we use these four specific patterns in Section “Joining patterns to find larger patterns” in detail.

Algorithm 1 presents the pseudo-code of our method. We elaborate on each key step of our method in subsequent sections. The algorithm takes a graph *G*, the number of nodes of the target motif *μ*, and the minimum acceptable motif frequency as input *α*. For each of the four basic building patterns, it first locates all subgraphs in *G* that are isomorphic to that pattern (Line 1). Let us denote the set of instances of the *i*th pattern (*i*∈ {1, 2, 3, 4}) with *S*_*i*_. In each set *S*_*i*_, it is possible to have overlapping subgraps. It then extracts the maximum set of edge-disjoint subgraphs in each set *S*_*i*_ (Line 2) (see Section “Finding MIS: Going from F1 to F2” for details). Let us denote the resulting set with *S* *i*′ for the *i*th pattern. Notice that the cardinalities of the sets *S*_*i*_ and *S* *i*′ are the *F*_1_ and *F*_2_ measures of the *i*th pattern respectively. The union of all the sets *S* *i*′ constitutes the current motif instances as well as the basic building pattern instances at this point (Line 3). The algorithm then iteratively grows the current motif set. At each iteration, it joins the current motif set with the basic building pattern set (Line 9). More specifically, a motif instance and a basic building pattern join if they share at least one edge. Joining two such subgraphs either creates a pattern which already exists in the current set (Line 10) or a new pattern (Line 12). At each iteration, after growing the current set, it filters the overlapping subgraphs to identify MIS for each pattern (Line 18). The algorithm removes all patterns with frequency lower than the user supplied cutoff (Line 21). It reports the frequent subgraphs that have as many edges as the target motif size (Line 23). The algorithm terminates when the current set can not be grown to have any other patterns which satisfy the target motif (i.e. each pattern in the current set is either larger than the target motif size or its frequency is lower than the user specified frequency).



### Joining patterns to find larger patterns

Here, we describe one join iteration of our method; the process of joining the subgraphs of current set of patterns with the subgraphs of the *basic building patterns* to construct larger patterns. At the end of the iteration, the resulting set of subgraphs becomes the current set of subgraphs for the next join iteration.

Recall that we join two subgraphs only if they share at least one edge. Joining two such subgraphs either yields a pattern that is isomorphic to one of the existing patterns or a new one. In the former case, we consider the set of subgraphs *S* isomorphic to that pattern. We check if the new subgraph is already in *S*. If it is in *S*, we discard it. Otherwise, we store it in *S*. In the latter case (i.e., the pattern is observed the first time), we save this as a new pattern and also keep the corresponding subgraph.

Notice that, although the subgraphs in *S* do not overlap prior to join, this may no longer hold after new subgraphs are inserted into *S*. At the end of each join iteration, we select the MIS for each pattern. We defer the discussion on how we do this to Section “Finding MIS: Going from F1 to F2”. We then remove the patterns with *F*2 values below the user supplied frequency threshold, *α*. This eliminates non-promising patterns, and thus, reduces the number of candidate patterns for the next join iteration. Using the *F*2 measure ensures that patterns maintain downward closure property. Thus, non-frequent patterns will never grow to yield frequent patterns.

**Why do we need different equivalence classes?** If the motif frequency is measured using *F*1, it is sufficient to join the subgraphs belonging to existing patterns with only those which belong to the same equivalence class of the simple pattern with two edges (see Fig. 2 a) to construct any larger pattern. This however is not true when *F*2 (or *F*3) is used to count the motif frequency. To understand the rationale behind this, recall that each equivalence class represents a set of disjoint isomorphic subgraphs. As a result, no two subgraphs from the same equivalence class join for they do not share any edges. Therefore we need more than one equivalence class to construct new and larger patterns.

Given that we need multiple patterns, next, we seek the answer to the following question: What is the smallest set of patterns which can be used to produce arbitrary large topologies by joining them? Here we outline the key steps of the proof that the four basic building patterns, presented in Fig. 2, suffice to construct any larger pattern. That said, we do not guarantee to find all copies of such patterns in the target network.

**Fig. 3:**
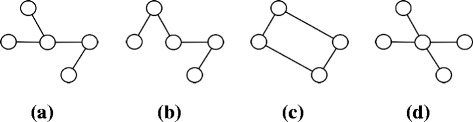
All patterns which can be constructed with four undirected edges. **a**, **b**, and **d** represent patterns with 4 edges and 5 nodes while **b** represents pattern with four nodes and four edges

Before we discuss our induction steps, we explain our strategy on a specific motif size of four to improve the clarity of the discussion on induction. Figure 3 shows all the possible patterns which can be constructed with undirected four edges. A careful inspection shows that each one is an overlapping combination of two of the basic building patterns. For instance, the pattern in Fig. 3 a can result from joining the basic pattern in Fig. 2 a with the basic pattern in Fig. 2 c. It is worth noting that we can construct some of the patterns in Fig. 3 by joining two different pairs of basic building patterns. This redundancy ensures we can still locate a specific pattern even if one of those pairs does not exist. Therefore, our method can construct any pattern with four edges from patterns with three or two edges.

We conduct our proof for the arbitrary pattern size by induction.

**Basis** The four basic patterns in Fig. 2 constitute all possible graph topologies with two or three edges.

**Induction step** We assume that our method can construct any pattern with up to *k* edges (*k*≥ 3). We next show that any pattern with *k*+1 edges can be constructed by joining a pattern with *k* edges with one of the basic building patterns.

Recall that the downward closure property states that those smaller patterns have at least as much frequency as the larger one according to *F*2 (see Theorem 1.1). This means that if a pattern with *k*+1 edges is frequent, then so is any of the *k* edge patterns obtained by removing an edge from that pattern.

Consider a graph *G* and a copy of a pattern *P*1 of size *k* edges in *G*, *S*_1_. Also, consider a copy of a pattern *P*2 with *k*+1 edges such that *P*2 contains *P*1 and one additional edge. Let us denote this additional edge with (*a, b*). We need to show that *P*2 can be obtained from *P*1 by joining it with at least one of the basic patterns.

**Fig. 4:**
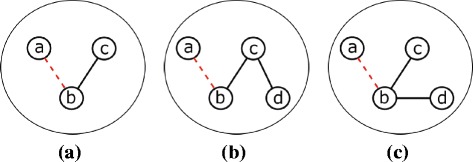
**a** A subgraph *S*_2_ in a hypothetical graph *G*. *S*_2_ is isomorphic to a pattern *P*2 of size *k*+1 edges. If we remove the additional edge (*a,b*) we obtain *S*_1_ which is isomorphic to *P*_1_ where *P*_1_⊂*P*_2_. Notice that *S*_1_ could have arbitrary *k*−1 edges rather than (*b,c*). Here we obtain *S*_2_ as a result of joining *S*_1_ with the subgraph {(*a,b*),(*b,c*)} which belongs to *M*1 equivalence class (see Fig. 2 a). **b** Failure to accomplish the join in (**a**), we seek to inspect *d* *e* *g*(*c*) and *d* *e* *g*(*b*) in *S*_1_. The first possibility is that *d* *e* *g*(*c*)>1. This means that the subgraph {(*b,c*),(*c,d*)} exists. We then can join *S*_1_ with the subgraph {(*a,b*),(*b,c*),(*c,d*)} which belongs to *M*4 equivalence class (see Fig. 2 d) to obtain *S*_2_ which is isomorphic to a pattern *P*2 of size *k*+1 edges. **c** The second possibility is that *d* *e* *g*(*b*)>1. This means that the subgraph {(*b,c*),(*b,d*)} exists. We then can join *S*_1_ with the subgraph {(*a,b*),(*b,c*),(*b,d*)} which belongs to *M*3 equivalence class (see Fig. 2 c) to obtain *S*_2_

Since both *P*1 and *P*2 are connected graphs, at least one of the two nodes *a* and *b* has an edge in *P*1. Without violating the generality of the proof, let us assume that *b* has an edge (*b,c*) in *P*1. Figure 4 a illustrates the two edges (*a, b*) and (*b, c*).

First, we consider using the basic pattern *M*1 in Fig. 2 a in the join operation. In this case, a copy of *M*1, {(*a,b*),(*b,c*)} will join with *S*_1_ having a common edge (*b,c*) which will result in the pattern *P*2 with *k*+1 edges. This join however occurs only if the subgraph {(*a,b*),(*b,c*)} is included in the *F*2 counts of *M*1 (i.e. within the chosen non-overlapping copies of *M*1).

If this condition fails, we consider the degrees of the two nodes *b* and *c* in pattern *P*1. We start with node *c*. Let us denote the degree of a node with function *d* *e* *g*() (e.g. *d* *e* *g*(*c*) is the degree of node *c* in pattern *P*1).

If *d* *e* *g*(*c*)>1, then *c* has at least one more edge on top of (*b,c*). Let us denote this edge with (*c, d*) (see Fig. 4 b). In this scenario, we join a copy of the motif *M*4 (Fig. 2 d), {(*a,b*),(*b,c*),(*c,d*)} (if this copy exists in the *F*2 count of *M*4) to obtain *P*2.

Finally, if *d* *e* *g*(*c*)=1, it is guaranteed that *d* *e* *g*(*b*)>1. This is because if both nodes *b* and *c* have degree one, *S*1 cannot be a connected subgraph. Let us denote one of the additional edges of *b* with (*b,d*) (see Fig. 4 c). In this case, we join the subgraph that isomorphic to the pattern *M*3, {(*a,b*),(*b,c*),(*b,d*)}, with *S*_1_ to obtain *P*2. We can do this if this copy exists in the *F*2 count of *M*3.

In summary, we conclude that any pattern *P*2 with *k*+1 edges can be constructed by joining a pattern *P*1 with *k* edges (or *k*−1 edges) and one of the basic building patterns to obtain the additional edge (or edges) if at least one of the many possible scenarios hold. We however cannot guarantee that the joins will find all of the instances of the *k*+1 edge pattern on the target graph.

Recall that as we aim to calculate the frequency of a given motif using *F*2, there is no self join of any pattern. Thus, the basic building patterns set is the smallest set of patterns as we can not construct one of those four patterns using the three other patterns. More specifically, this means that we can not use only one of those four basic building patterns to construct larger patterns by joining pairs of subgraphs belong to that pattern’s equivalence class. This is because if we join the embeddings of a single motif topology (such as the first pattern in Fig. 2 a) we cannot get any larger pattern as they do not share any edge(s).

### Finding MIS: Going from F1 to F2

Here, we explain how we compute the *F*2 frequency for a given pattern. We use two algorithms for this purpose. We explain why we have two separate algorithms later in this section after describing the two algorithms. The first one is a heuristic used in the literature [16]. This algorithm constructs a new graph, called the *overlap graph* for each pattern as follows. Each node in the overlap graph of a pattern denotes an embedding of that pattern in the target graph. We add an edge between two nodes of the overlap graph if the corresponding embeddings represented by those nodes overlap in the original graph. Once the overlap graph is constructed, the algorithm starts by selecting the node with the minimum degree (i.e. overlaps with the minimum number of embeddings) in the overlap graph. We include the subgraph represented by this node in the edge-disjoint set. We then delete that node along with all of its neighboring nodes in the overlap graph. We update the degree of the neighbors of the deleted nodes. We repeat this process of picking the smallest degree node and shrinking the overlap graph until the overlap graph is empty.

The algorithm described above works well for patterns with small number of embeddings. It however becomes computationally impractical as the number of embeddings of the underlying pattern gets large. This is because both constructing the overlap graph (particularly identifying its edges) and updating it are computationally expensive tasks. Therefore, we use this algorithm for all patterns except for the basic building patterns (where number of embeddings are often too large).

**Fig. 5:**
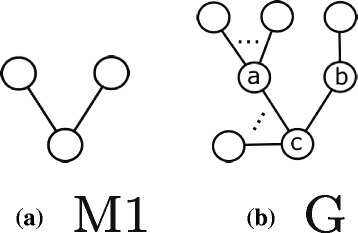
**a** One of the basic building patterns. **b** A hypothetical graph that contains subgraphs isomorphic to the pattern M1 in (**a**)

The second algorithm addresses the scalability issue of the the first one. This scalability issue is imposed by the expensive task of calculating the degree of each node in the overlap graph (i.e. the number of overlaps of each embedding). Recall from the previous algorithm that this number is considered as a loss value when selecting the node (i.e. embedding) with minimum degree (i.e. number of overlaps) to include in the final MIS of the pattern under consideration. Briefly, the second algorithm we introduce here avoids the expensive task of calculating number of overlaps for each embedding. The algorithm performs this by *algebraically* computing such numbers instead of performing actual overlapping tests. Once we compute node degrees of the overlap graph, this algorithm selects the disjoint embeddings the same way as the former algorithm described before. More specifically, the algorithm selects the node with the minimum degree and includes its corresponding embedding in the final MIS. It then removes neighboring nodes to that node from the overlap graph. It repeats this process until the overlap graph is empty. Next, we explain how we compute the degree of a node in the overlap graph for the pattern *M*1 in Fig. 2 a. Our computation is similar for the other three basic building patterns, yet tailored towards their specific topologies (derivation is shown in Additional file 1: Appendix). Figure 5 shows a hypothetical subgraph *S*_1_={ (*a,c*), (*b,c*)} in the input graph *G* which is isomorphic to *M*1. This subgraph is represented by a node in the overlap graph of *M*1’s embeddings. Let us denote the degree of a node in the original graph *G* with function *d*() (e.g. *d*(*v*_*i*_) is the degree of node *v*_*i*_). Another embedding of *M*1 in *G* overlaps with *S*_1_ only if it contains the edge (*a,c*), or (*b,c*). Any edge in *G* connected to the middle node *c* forms two overlapping embeddings, one with the subgraph that has edge the (*a,c*) and the other with the subgraph that has the edge (*b,c*). We exclude the edges belong to *S*_1_ (i.e. the embedding we want to calculate its number of overlaps) itself from the potential edges of *G* that considered in the overlapping embeddings with *S*_1_. Thus, by excluding the two edges (*a,c*) and (*b,c*) from *c*’s degree, node *c* yields 2 × (*d*(*c*) - 2) overlaps. In addition, any edge that belongs to node *a* forms an embedding when combined with the edge (*a,c*). Excluding the edge (*a,c*), node *a* yields *d*(*a*) - 1 overlaps. Similarly, node *b* produces *d*(*b*) - 1 overlaps. Thus, the total number of overlaps for the embedding *S*_1_ = { (*a,c*), (*b,c*)} combined from edges of its three nodes { (*a,b,c*)} is

$$ 2(d(c) - 2) + d(a) - 1 + d(b) - 1 = 2d(c) + d(a) + d(b) - 6 $$

Notice that unlike the first algorithm, the second one requires a unique derivation for each pattern. Thus, we apply it only to the basic building patterns, for their topologies do not depend on the input graph. Also, it is worth noting that typically the basic building blocks have much larger number of embeddings as compared to the patterns derived by joining them. Thus, the efficiency of the second algorithm is needed for them more than the patterns obtained in subsequent iterations (see experimental results).

**Fig. 6:**
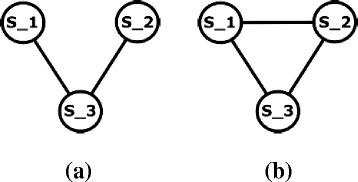
**a** The overlap graph of the pattern in Fig. 1 c based on *F*_2_ measure of this pattern in the graph in Fig. 1 a. **b** The overlap graph of the same pattern based on *F*_3_ measure

To adapt our method to count non-overlapping embeddings of each pattern according to *F*3 instead of *F*2, we only need to change how we calculate the MIS of this pattern. More specifically, we change the criteria which states that “two subgraphs overlap if they share at least one edge” to “two subgraphs overlap if they share at least one node” (see Section “Definitions and notation”). This will result in changing the overlap graph constructed using the first method we explain in this section. In addition, it will also have slight change in calculating the total number of overlap of each embedding using the second method we discuss in this section. Practically, we expect the overlap graph to be denser when we use the *F*3 measure as compared to that for the *F*2 measure. To illustrate this, consider the graph *G* in Fig. 1 a and the pattern in Fig. 1 c. This patter have 3 embeddings in *G* which are *S*_1_, *S*_2_, and *S*_3_ defined by the set of edges {(a,b), (a,c), (b,c), (b,e)}, {(e,f), (f,g), (e,g), (e,d)}, {(e,f), (f,g), (e,g), (b,e)} respectively. Figure 6 a and Fig. 6 b represent the overlap graph of this pattern based on *F*2 and *F*3 measures respectively.

### Accelerating our algorithm through efficient filters

Recall that at each iteration, our algorithm generates new subgraphs. For each of these subgraphs, it checks if this subgraph is isomorphic to one of the patterns constructed till that iteration. Isomorphism test is a computationally expensive task. Next, we describe how we avoid a large fraction of these tests.

We develop two canonical labeling strategies for patterns. Canonical labeling assigns unique labels to the nodes of a given pattern [27]. If two patterns are isomorphic, then they have the same canonical labeling. The inverse is however not true. Unlike isomorphism test, comparing the canonical labeling is a trivial task. Following from this observation, when we construct a new subgraph, we first compare its canonical labeling to those of existing patterns. We then limit the costly isomorphism test to only those patterns which have the same canonical labeling as the new subgraph.

The first canonical labeling counts the degree (i.e. number of incident edges) of each node in the given pattern. It then sorts those degrees and keeps them as a vector we call the *degree vector*. If two patterns have different degree vectors, then they are guaranteed to have different topologies. Despite its simplicity, this labeling filters out a large fraction of patterns. To test its efficiency, we have tested it on random graphs generated using Barabási −Albert model [28]. We generate 1000 pairs of graphs where each pair is non-isomorphic and have the same number of nodes and edges. The degree vector successfully filters 85 % of the 1000 experiments.

The second canonical labeling extends on the first one. It was first introduced by [29]. Consider a pattern *P*=(*V, E*). Let us define the distance between two nodes *v*_*i*_, *v*_*j*_∈*V* as the number of edges on the shortest path that connects *v*_*i*_ and *v*_*j*_ and denote it with *x*_*ij*_. Let us define the diameter of *P* as the maximum distance between any two nodes, and denote it with *x*. Using this notation, we assign label to node *v*_*i*_ as: $\sum _{j}^{j \in V} 2^{x-x_{ij}-d(v_{j})}$. Once we compute the labels of all the nodes in the given pattern, we sort them. We call the resulting vector the nodes vector. Similar to the first labeling above, two isomorphic graphs are guaranteed to yield the same labeling. We compute and compare the nodes vector with only the patterns which cannot be eliminated using the first canonical labeling. We then consider the patterns with identical canonical labels for graph isomorphism.

### Complexity analysis

Here we analyze the complexity of our method. We refer to Algorithm 1 as we discuss the steps of our method. For each steep, we explain its complexity. We then summarize the complexity of all steps to denote the overall complexity of our method. These steps are

- **Find all subgraphs isomorphic to each of the four basic patterns (Line 1)**: In this step, we analyze each of the four basic patterns separately since they have different topologies. For the pattern *M*1 in Fig. 2 a, to get all subgraphs isomorphic to this pattern, we consider all edges connected to each node in the underlying network. We select any two edges combination connected to every node. Here, we denote the degree of a node with function *d*() (e.g. *d*(*v*_*i*_) is the degree of node *v*_*i*_). Thus, the complexity of collecting subgraphs that are isomorphic to *M*1 is ${\sum \nolimits }_{v_{i} \in V} {{d(v_{i})}\choose {2}}$. Similarly, for the pattern *M*3 in Fig. 2 c, we select any three edges combination connected to each node in *G*. Thus, the complexity of constructing subgraphs which are isomorphic to *M*3 is ${\sum \nolimits }_{v_{i} \in V} {{d(v_{i})}\choose {3}}$. For the pattern *M*2 in Fig. 2 b, we consider each edge *e*_*ij*_ in *G* with two nodes *v*_*i*_ and *v*_*j*_. We collect edges of both nodes. We then select one edge connected to *v*_*i*_ and one edge connected to *v*_*j*_ (on the condition that these two edges are connected from the other end) along with *e*_*ij*_ to form a subgraph isomorphic with *M*2. Thus, the complexity of constructing subgraphs that are isomorphic to *M*3 is ${\sum \nolimits }_{e_{ij} \in E} d(v_{i}) d(v_{j})$. Similarly to *M*2, we perform the same operation to get isomorphic subgraphs to the pattern *M*4 in Fig. 2 d. Only this time we make sure that the two edges belong to *v*_*i*_ and *v*_*j*_ are not connected with each other from the other end. Thus, the complexity of constructing subgraphs that are isomorphic to *M*4 is ${\sum \nolimits }_{e_{ij} \in E} d(v_{i}) d(v_{j})$. Collectively, the complexity of performing this step is $\mathcal {O}({\sum \nolimits }_{v_{i} \in V} d(v_{i})^{3} + {\sum \nolimits }_{e_{ij} \in E} d(v_{i}) d(v_{j}))$. Notice that, theoretically, the worst case scenario happens when $d(v_{i}) = \mathcal {O}(n)$. In this scenario, the complexity of this step becomes $\mathcal {O}(n^{4})$.
- **Extract maximum disjoint set for basic patterns (Line 2)**: In this step, we use the algebraic algorithm described in Section “Finding MIS: Going from F1 to F2” (second one) to calculate the number of overlaps of each subgraph belonging to each pattern equivalence class. This process takes constant time. We calculate this algebraic equations as we construct subgraphs in the previous step. We then sort those subgraphs within each equivalence class in decreasing order of their number of overlaps. This process has complexity equal to $\mathcal {O}(mlog(m))$ where *m* is the number of subgraphs in each equivalence class. Recall from previous step that this number is $\mathcal {O}\left ({\sum \nolimits }_{v_{i} \in V} d(v_{i})^{3} + {\sum \nolimits }_{e_{ij} \in E} d(v_{i}) d(v_{j})\right)$. Thus, the complexity of this step is $\mathcal {O}\left (\left ({\sum \nolimits }_{v_{i} \in V} d(v_{i})^{3}\right)\right.$ $ log\left ({\sum \nolimits }_{v_{i} \in V} d(v_{i})^{3}\right) + \left ({\sum \nolimits }_{e_{ij} \in E} d(v_{i}) d(v_{j})\right) $ $\left.log\left ({\sum \nolimits }_{e_{ij} \in E} d(v_{i}) d(v_{j})\right)\right)$.
- **Join Iterations (Lines 5–27)**: In this step, we analyze the complexity of one join iteration. We then summarize the complexity of all join iterations. Let us denote the number of current patterns in iteration *i* with *x*_*i*_. Notice that, for the first iteration *x*_*i*_=4. Recall that in each join iteration, we increase the size of each of the current patterns with one or two edges. In addition, the patterns of the first join iteration are at least of size 2. Thus, the size (i.e. number of edges) of each of the current patterns in iteration *i* is at least *i*+2. The number of subgraphs isomorphic to each of the current patterns is at most $\frac {|E|}{i+2}$ since they are non-overlapping subgraphs. Recall that the subgraphs of the basic patterns are non-overlapping within each pattern. Thus, the number of subgraphs of the patterns *M*1, *M*2, *M*3, and *M*4 are $\frac {|E|}{2}$, $\frac {|E|}{3}$, $\frac {|E|}{3}$, and $\frac {|E|}{3}$ respectively. Collectively, the number of subgraphs of the basic patterns is $\mathcal {O} (|E|)$.
  In the join iteration, we start by joining subgraphs of current patterns with the subgraphs of the basic patterns (Lines 6–9). Thus, the total number of joins we perform at iteration *i* is $\mathcal {O}\left (|E| \frac {|E|}{i+2} x_{i}\right)$. For each join, we compare the resulting subgraph against all patterns (Line 10). Recall that, we use filters to avoid this costly isomorphism check (see Section “Accelerating our algorithm through efficient filters”). Thus, the complexity of this operation is $\mathcal {O} (x_{i})$. If this subgraph is isomorphic to one on the current patterns, we check whether this subgraph is a duplicate of one of the subgraphs which already exists in this equivalence class (Line 11). We search an indexed list of those subgraphs in $\mathcal {O} \left (log\left (\frac {|E|}{i+2}\right)\right)$. Collectively, we obtain the complexity of performing all joins at iteration *i* by multiplying the three complexities above and get $\left (|{E}| \frac {|E|}{i+2} x_{i} x_{i} log\left (\frac {|E|}{i+2}\right)\right), \mathrm {which~equals}\mathcal {O}\left ({x_{i}^{2}} \frac {|E|^{2}}{i+2} log\left (\frac {|E|}{i+2}\right)\right)$.
  Upon completing all join operations, our algorithm extracts the MIS for each pattern (Line 18) using the overlap graph algorithm described in Section “Finding MIS: Going from F1 to F2” (first one). Notice that we perform this operation for the new set of patterns, *x*_*i*+1_ (current patterns of next iteration) for which the number of patterns is at most $\frac {|E|}{i+3}$ (This is because each pattern is of size *i*+3 and no two patterns overlap). For each pattern, we collect the overlapped subgraphs of each subgraph in $\mathcal {O}\left (\left (\frac {|E|}{i+3}\right)^{2}\right)$. We then sort the subgraphs in decreasing order of their number of overlaps in $\mathcal {O}\left (\frac {|E|}{i+3} log\left (\frac {|E|}{i+3}\right)\right)$ time. Thus we extract the MIS for all patterns in $\mathcal {O}\left (x_{i+1} \left (\frac {|E|}{i+3}\right)^{3} log\left (\frac {|E|}{i+3}\right)\right)$.
  Finally, we check each resulting pattern (Line 19–25) and delete it if its frequency is less than the threshold *α*. We perform this step in $\mathcal {O}(x_{i+1})$ time.
  Recall that in each join iteration, we increase the size of each of the current patterns with one or two edges. Also recall that we start the with patterns of at least of size 2. Thus, total number of join iterations we perform until we reach to all patterns are at least of the target motif size is *μ*−2. Thus, the complexity of all join iterations is $\mathcal {O}\left (\sum \limits _{i=1}^{\mu - 2} \left ({x_{i}^{2}} \frac {|E|^{2}}{i+2} log\left (\frac {|E|}{i+2}\right) + x_{i+1} \left (\frac {|E|}{i+3}\right)^{3} log\left (\frac {|E|}{i+3}\right)+ x_{i+1}\right)\right)~\mathrm {or~simply} \mathcal {O}\left (\sum \limits _{i=1}^{\mu - 2} \left [x_{i} \frac {|E|^{2}}{i} log\left (\frac {|E|}{i+2}\right)\right ] \left [x_{i} + \frac {|E|}{i^{2}}\right ] + x_{i+1}\right)$

In summary, the complexity of our method considering all the previous steps is

$$\begin{array}{*{20}l} & \mathcal{O}\left(\left({\sum\nolimits}_{v_{i} \in V} d(v_{i})^{3}\right) \left(1+ log\left({\sum\nolimits}_{v_{i} \in V} d(v_{i})^{3}\right)\right)\right. \\ &\quad+\! \left({\sum\nolimits}_{e_{ij} \in E} d(v_{i}) d(v_{j})\right) \left(1+ log\left({\sum\nolimits}_{e_{ij} \in E} d(v_{i}) d(v_{j})\right)\right) \\ &\quad\left. + \sum\limits_{i=1}^{\mu - 2} \left(\left[x_{i} \frac{|E|^{2}}{i} log\left(\frac{|E|}{i+2}\right)\right] \left[x_{i} + \frac{|E|}{i^{2}}\right] + x_{i+1}\right)\right) \end{array} $$

Notice that *x*_*i*_ here depends significantly on the topology and the density of the given network *G*. To the best of our knowledge, there is no closed formula that calculates *x*_*i*_ (i.e. the number of unique topologies of certain size in a given graph G).

## Results and discussion

In this section, we experimentally evaluate the performance of our motif discovery algorithm on synthetic and real graphs (Section “Evaluation of running time”). We measure the running time and accuracy of our algorithm. We compare our algorithm to two state of the art algorithms, FSG [25] and SUBDUE [15] (Section “Comparison with existing methods”). We evaluate the statistical significance of the most abundant motif in each of the real graph (Section “Evaluation of statistical significance”). We present a case study of the motifs identified by our method on Human herpesvirus PPI network (Section “Case study on Human herpesvirus”). In all of our experiments, we report the motif frequency using the *F*2 measure.

**Data set** We use real and synthetic datasets in our experiments. The real graphs are the PPI networks of seven organisms taken from the MINT database [30] (see Table 1 for details). We first remove the nodes and edges of these graphs which are guaranteed to not be a part of the motif to be found. To do that, we filter a subset of the nodes of each network as follows. We first identify connected subgraphs of each graph. Let us denote the size of the motif we aim to find with *μ*. We remove the connected subgraphs with less than *μ* nodes. Table 1 lists these networks and their sizes after filtering them for *μ*=5 (which is the smallest motif size in all of our experiments).

**Table 1:** The size (number of Proteins and interactions) of the PPI networks selected from the MINT database

Network name	Network	Number of	Number of
	code	proteins	interactions
Human herpesvirus8	hhv-8	48	82
Campylobacter jejuni	cje	109	117
Treponema pallidum	tpa	108	173
Rattus norvegicus	rno	535	643
Helicobacter pylori	hpy	717	1472
Escherichia coli	eco	616	1561
Plasmodium falciparum	pfa	1221	2577

In addition to the real dataset, we construct synthetic graphs. The purpose of having synthetic dataset is to systematically evaluate our method by varying network characteristics (network size and density) in a controlled environment. We build this dataset using the Barabási −Albert model [28] for it captures the connectivity patterns of real networks [31–33]. Moreover, this model has been frequently used in the literature to simulate real networks.

**Implementation and environment** We implement our algorithm in C++ and perform experiments on a computer equipped with AMD Opteron(tm) Processor 1.4 GHz CPU, 500 GBs of main memory running Linux operating system.

### Evaluation of running time

In this experiment, we evaluate the running time of our motif discovery algorithm. Our goal here is to observe the effect of varying parameters; graph size, graph density, and motif size on the running time of our algorithm.

#### Effect of graph and motif size

We evaluate the running time of our method under varying graph and motif sizes using both synthetic and real datasets.

##### Results on synthetic graphs

**Fig. 7:**
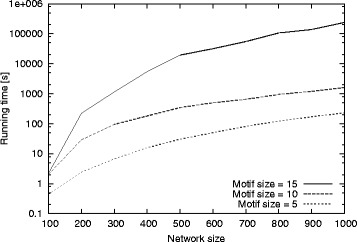
The total running time of our method for varying graph size and motif sizes (number of nodes). Motif size varies from 5 to 15. The x-axis shows the input graph sizes varying from 100 to 1000. The y-axis shows the total running time in seconds

We generate synthetic graphs of varying size (i.e. number of nodes) from 100 to 1000 at increments of 100. We fix the graph density to two edges per node on the average (i.e., mean node degree is set to four). We set the minimum desired motif frequency, *α*= 10. We run experiments for motif sizes *μ*= 5, 10, and 15 and report the running time. Figure 7 presents the results.

The results demonstrate that our method scales well with growing graph and motif sizes. The running time grows with increasing graph and motif sizes, yet it remains practical for very large graphs. For motif sizes of 5 and 10, it runs in only several minutes even for the largest input graph. As the motif size grows, the cost increases. However, our method can identify very large motifs in a little over a day for massive networks. We observe that the motif size has more influence on the performance of our method than the input graph size. This is because the number of alternative motif topologies grow exponentially with the motif size. This is an inherent characteristic of the underlying computational problem. However, even when the motif size is 15 our method remains to have a practical running time.

##### Results on real graphs

**Fig. 8:**
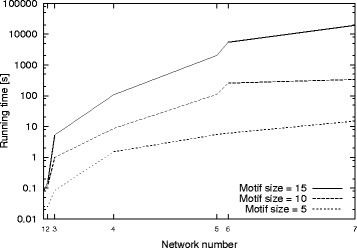
The total running time of our method for the real PPI networks. Network numbers 1 to 7 on the x-axis correspond to hhv-8, cje, tpa, rno, hpy, eco, and pfa PPI networks respectively. The positions of the PPI networks on the x-axis indicate the sizes of the input graphs (see Table 1). The y-axis shows the running time in seconds

Next, we test our method on real dataset. We set the minimum desired motif frequency, *α*= 5. We run experiments for motif sizes *μ*= 5, 10, and 15 and report the running time. Figure 8 presents the results. Similar to the synthetic dataset results, our method scales to large graph and motif sizes on the real dataset. Note that the number of alternative motif topologies grows exponentially with the motif size. Furthermore, the cost of subgraph isomorphiosm also grows exponentially with the motif size. Despite these two major complicating factors, the running time of our method increases only by about an order of magnitude when we increase the motif size by five. Finally, the parallel between these results and those in Fig. 7 suggests that synthetic graphs generated by Barabási −Albert model have similar structural properties as the real PPI graphs.

#### Effect of graph size and density

**Fig. 9:**
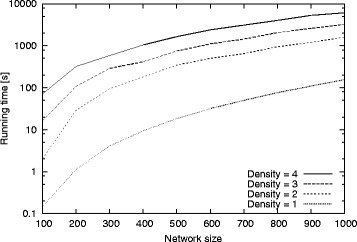
The total running time of our method for the synthetic graphs with different graph sizes (number of nodes) and varying graph densities from 1 to 4. The x-axis shows the input graph sizes. The y-axis shows the total running time in seconds

Here, we evaluate the effect of varying input graph size and density on the running time of our algorithm. We use synthetic dataset in order to control the graph density in this experiment. We generate synthetic graphs varying network size from 100 to 1000 at increments of 100. We set the desired motif frequency *α*= 5 and the motif size *μ*= 10. We vary graph density from one to four which covers broad range of biological networks [34]. For each input graph and density value, we report the total running time. Figure 9 presents the results.

We observe that the running time increases with growing graph density. As the graph density increases, the number of alternative embeddings of a given motif grows as well. This also increases the number of overlapping subgraph pairs, which in turn increases the cost of finding MIS for each pattern to calculate its *F*2 frequency (see Section “Finding MIS: Going from F1 to F2”). Despite these major complications inherent in the nature of the motif counting problem, our method remains scalable with respect to growing density. These results suggest that our method is reliable and computationally feasible for a broad range of networks with different sizes and densities.

### Comparison with existing methods

Here, we compare our method against two methods in the literature which are tailored towards a problem similar to the one considered in this paper, namely SUBDUE and FSG. We measure the running time and accuracy. We compute accuracy in terms of three parameters, the number of unique motifs found, the average frequency per motif in the target graph, and the frequency of the most abundant motif.

Of these two methods, for SUBDUE, we only report the accuracy of the result as we observe that for most datasets and motif sizes, SUBDUE fails to identify motifs (results shown later in this section). For FSG, we only report the running time. This is because FSG finds motifs in multiple graphs, limited to at most one embedding per graph. In other words, it cannot find multiple embeddings of the same motif in a single graph. Therefore, FSG would yield very low accuracy when applied to a single graph. In the rest of the paper, we will refer to our method as MD (Motif Discovery) for simplicity.

#### Comparison with SUBDUE

**Fig. 10:**
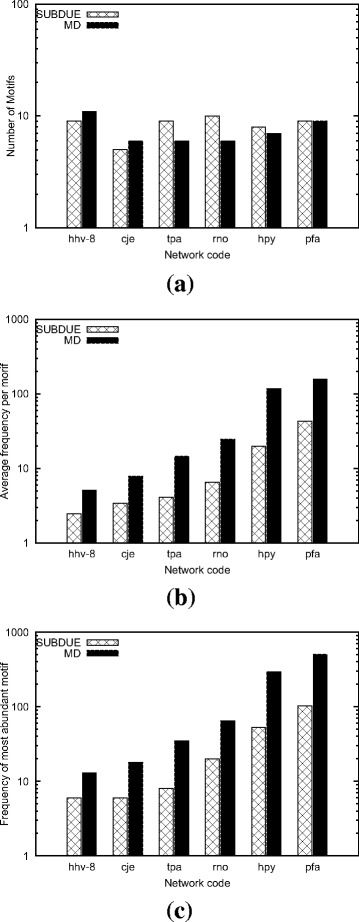
The accuracy of our method (MD) and SUBDUE in terms of three measures **a** the number of unique motif topologies found, **b** the average frequency per motif in the target graph, and **c** the frequency of the most abundant motif. Results are for the motif size *μ*= 5 on the real dataset (see Table 1)

**Fig. 11:**
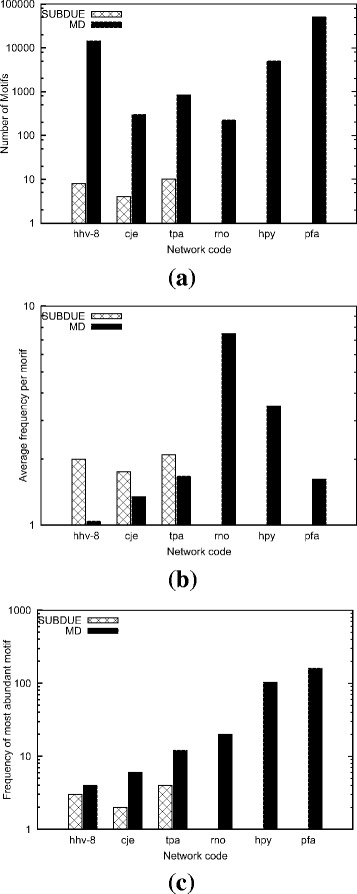
The accuracy of our method (MD) and SUBDUE in terms of three measures **a** the number of unique motif topologies found, **b** the average frequency per motif in the target graph, and **c** the frequency of the most abundant motif. Results are for the motif size *μ*= 10 on the real dataset (see Table 1)

**Fig. 12:**
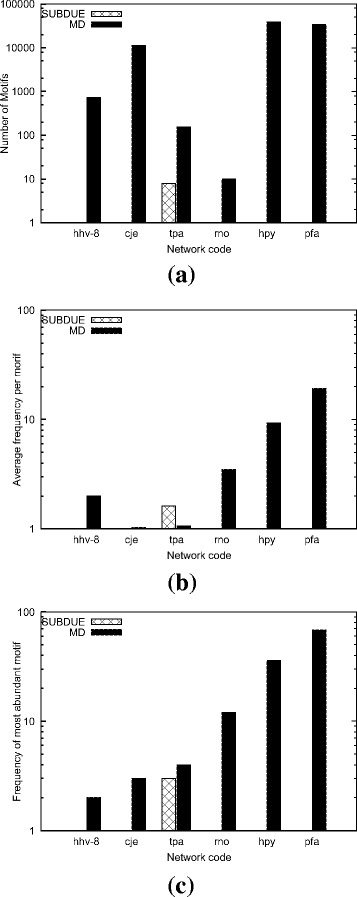
The accuracy of our method (MD) and SUBDUE in terms of three measures **a** the number of unique motif topologies found, **b** the average frequency per motif in the target graph, and **c** the frequency of the most abundant motif. Results are for the motif size *μ*= 15 on the real dataset (see Table 1)

In this experiment, we analyze the effect of varying input graph and motif sizes on the accuracy of our method as compared to that of SUBDUE. We use real dataset in this experiment (see Table 1). SUBDUE does not allow the user to set a minimum allowable motif frequency parameter. It finds all subgraph topologies of a given size even for those subgraphs that appear only once. Due to this limitation of SUBDUE, to have a fair comparison, we set *α*= 1 for our method as well. We follow our earlier definition (see “Definitions and notation”), and use motif size *μ* to denote the number of nodes in the given motif topology. We run both methods on each input graph using motif sizes *μ*= 5, 10, and 15. We report the accuracy of our method as well as SUBDUE. Figures 10, 11, and 12 present the results of *μ*= 5, 10, and 15 respectively.

Our results for *μ*= 5 (Fig. 10) demonstrate that both methods identify similar number of unique motifs, yet our method outperforms SUBDUE significantly in terms of the average frequency per motif in all cases (see Fig. 10 b). When we focus on the most abundant topology of each method, we observe a similar pattern; our method always finds patterns with much higher frequency than SUBDUE in all the experiments (see Fig. 10 c). It is worth nothing that motif discovery problem gets exponentially harder with growing motif size. As a result, we expect most algorithms tailored for motif identification to perform well for small motif sizes such as *μ*= 5. Next, we observe how our method and SUBDUE perform for large values of *μ*.

As we grow the motif size to *μ*= 10 (Fig. 11), the results suggest that the gap between our method and SUBDUE grows rapidly in terms all three accuracy measures. More importantly, the results also show that in half of the cases, particularity where the input graph size is large, SUBDUE could not find any motifs while our method continue to locate patterns with high frequency. For example, our method was capable of finding motif topologies with frequency over 100 while SUBDUE could not locate any motif (see Fig. 11 c).

For few cases (see Fig. 11 b), (hhv-8, cje, and tpa), the average frequency per motif of SUBDUE is slightly higher than that of our method. This is because, we set the minimum frequency *α*= 1. Our method locates many topologies which exist only once while SUBDUE fails to locate them. For example, our algorithm finds thousands of unique motif topologies while subdue outputs only 8 motif topologies for the hhv-8 organism (see Fig. 11 a). As a result, these unique topologies pull the average frequency down. That said, Fig. 11 c confirms that our method can identify motifs which are more frequent than those found by SUBDUE even for those organisms.

As we further increase the motif size to *μ*= 15 (Fig. 12), the significance of our method becomes more prevalent. We observe that SUBDUE could not find any motifs in any of the graphs accept for tpa’s PPI network. On the other hand, our algorithm not only identifies a massive number of patterns (see Fig. 12 a), but also some of these patterns have very large frequencies (see Fig. 12 c).

In summary, the results demonstrate that our method scales to large input graph and motif sizes and continue to locate patterns with high frequency for a broad range of motif and input graph sizes while SUBDUE fails to do so.

#### Comparison with FSG

**Fig. 13:**
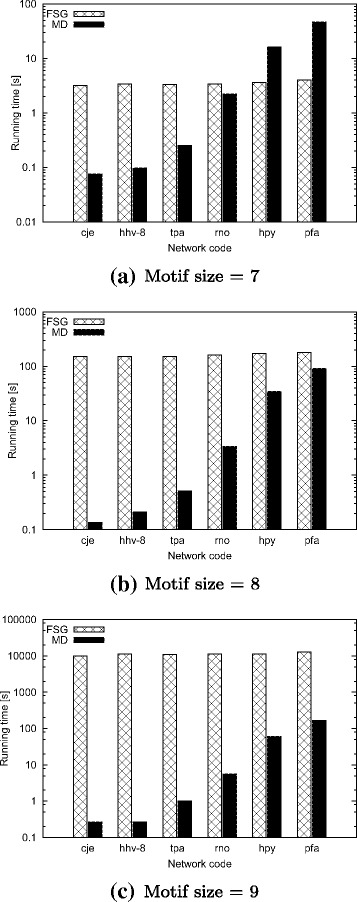
The total running time of our method (MD) and FSG for the real PPI networks (see Table 1). The y-axis shows the running time in seconds for three motif sizes; **a**. motif size = 7, **b**. motif size =8, and **c**. motif size =9

In this experiment, we compare the effect of different input graph and motif sizes to the running time of our algorithm and that of FSG. We use real dataset in this experiment (see Table 1). FSG method requires multiple graphs as input. It defines the frequency of the motif topology as number of different graphs that this motif appears within. Since our method operate on one input graph, we set the desired motif frequency *α*= 1 to be consistent with FSG. FSG defines motif size as the number of edges in the given motif. To be consistent with FSG, we use *μ* to denote the number of edges in the motif in this experiment. We run both methods on each input graph using motif sizes *μ*= 7, 8, and 9. We report the running time of our method (MD) as well as FSG. We do not run experiments for *μ*> 9 as FSG fails to scale to large motif sizes unlike our method. Figure 13 presents the results.

We observe that our method (MD) is orders of magnitude faster than FSG, particularly in large motif sizes. The running time of our method increases slowly with both motif size and the graph size. On the other hand, the running time of FSG increases slowly with the input graph size, but very rapidly with the motif size. Only for a few cases of small motif sizes (i.e ≤7 edges) FSG performs better than our method. This is due the overhead of calculating F2 for the basic building patterns where number of overlapped embeddings is huge. That said, the running time difference in those cases are negligible. These results suggest that our method outperforms FSG in terms of running time for a broad range of input real biological networks with different sizes. This performance advantage is further magnified by the fact that our method can find multiple embeddings of each motif while FSG finds only one. The two main reasons behind the fact that our method is significantly faster than FSG is that our method (i) does not calculate the frequency of the each new pattern by locating the copies of this pattern in the network using subgraph isomorphism as FSG does, and (ii) it ensures that every generated pattern exists at least once in the underlying graph.

### Evaluation of statistical significance

In this experiment, we evaluate the statistical significance of the most abundant motif identified by our method in each of the six PPI networks (see Table 1). We compute the statistical significance of the abundance of the most frequent motif of a given size in two alternative approaches. Each of these two approaches measures a different aspect of the significance.

- The first approach measures the statistical significance of the frequency of most abundant motif with respect to the abundances of all motifs with the same size in the same graph. More specifically, given a target graph *G*=(*V, E*) and motif size *μ*, we first find all motifs of size *μ* in *G*. Assume that there are totally *m* such motifs. Let us denote the frequency of these motifs with *x*_1_, *x*_2_, …, *x*_*m*_, with *x*_1_ being the largest among all. Let us denote the mean and standard deviation of these *m* frequency values with $\bar {x}$ and *σ*. We report the *z*-score of the frequency of the most abundant motif as $\frac {x_{1} - \bar {x}}{\sigma }$.
- The second approach measures the statistical significance of the frequency of the most abundant motif in the original graph with respect to those in the random ensemble of graphs of the same size and degree distributions. More specifically, given a target graph *G*=(*V, E*) and motif size *μ*, let us denote the frequency of the most abundant motif of this size in *G* with *x*. We construct a set of *n* random networks from *G* through *degree preserved edge shuffling* [35, 36]. Note that degree preserved edge shuffling is an iterative technique, which is often used in the literature to construct random network topologies with same size and degrees as a given target graph *G*=(*V, E*). At each iteration of this technique, we randomly pick two edges from *E*. Let us denote these edges with (*v*_1_, *v*_2_) and (*u*_1_, *u*_2_), where *v*_1_, *v*_2_, *u*_1_, *u*_2_∈*V*. We remove these two edges from *E* and insert two new edges (*v*_1_, *u*_2_) and (*u*_1_, *v*_2_). This way as the network topology evolves randomly, we ensure that the degrees of all the nodes remain unchanged. We repeat these iterations large number of times (exactly 10 ×|*E*| times) to randomize the entire network. Using the strategy above, we generate 100 random graphs, denoted with *G*_1_, *G*_2_, …, *G*_100_. For each random graph *G*_*i*_, we measure the frequency of the most abundant motif of size *μ*. Let us denote this number as *x*_*i*_. Let us denote the mean and standard deviation of these 100 frequency values with $\bar {x}$ and *σ*. We report the *z*-score of the frequency of the most abundant motif as $\frac {x - \bar {x}}{\sigma }$.

For both of the approaches above, we assume that a *z*-score above 2 or below -2 implies high statistical significance (i.e., two standard deviations away from the mean). The larger the magnitude of z-score is, the more significant the result is. Tables 2 and 3 present the *z*-score for each of the six PPI network and three motif size (*μ*= 5, 10, 15) combinations using the first and the second approach described above respectively.

**Table 2:** The *z*-scores that represent signifncance of the most abundant motif aginast other motifs in in the same network in each PPI network usig three motif size

Network code	Motif size = 5	Motif size = 10	Motif size = 15
hhv-8	1.52	14.00	4.67
cje	1.41	5.53	12.12
tpa	1.45	7.19	3.36
rno	1.58	4.31	9.74
hpy	1.54	13.70	9.003
pfa	1.87	35.32	7.43

**Table 3:** The *z*-scores that represent signifncance of the most abundant motif aginast most abundant motifs in 100 random networks in each PPI network usig three motif sizes

Network code	Motif size = 5	Motif size = 10	Motif size = 15
hhv-8	2.79	-0.54	-2.83
cje	2.32	0.99	-0.82
tpa	3.21	5.27	2.83
rno	-0.49	-4.02	-4.83
hpy	22.42	8.61	6.15
pfa	10.53	5.16	4.80

Table 2 suggests that, for small motif size (i.e. *μ*= 5), the most abundant motif is not significantly more frequent than other motifs of the same size. However, as motifs get large in size (i.e. *μ*= 10 and 15), the gap between the frequency of the most abundant motif and the rest of the motifs becomes highly significant. This implies that larger motifs characterize topological properties of PPI networks better than small motifs. This is because when motif size is small different motifs have similar frequency values, and this cannot be statistically different in abundance than each other. On the other hand, for large motif size, although the number of unique motif topologies is large, they vary a lot in their abundances; the most frequent one gets significantly more abundant than the rest.

Table 3 shows that, for most of the PPI network and motif size combinations, the most abundant motif is highly over-represented in the original network compared to random networks. In three cases (*Rattus norvegicus*, *μ*= 10 and 15, and *Human herpesvirus8*, *μ*= 15), we observe that the most abundant is significantly under-represented. These results demonstrate that the motif abundance in PPI networks is not random for nearly all combinations we tested. Thus, studying these structures has great potential to help understand how these networks function. Among the six PPI networks, *Rattus norvegicus* stands out to be the one with consistently under-represented or random motif abundance. The PPI of *Helicobacter pylori* consistently has the most significant motif abundance for all motif sizes. This indicates that the interactions in this network follow a regular pattern repeating themselves at different locations of the network. Finally, notice that the two z-score values reported in Tables 2 and 3 do not follow the same pattern (that is a high z-score according to one measure does not imply a high value for the other). This implies that the frequencies of different motifs (i.e., including the ones which are not most abundant) in these PPIs differ from those in random networks. In other words, the PPI networks topologically deviate from random networks.

### Case study on Human herpesvirus

**Fig. 14:**
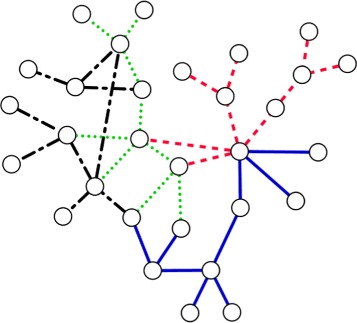
The organization of the four isomorphic subgraphs of 10 nodes in the hhv-8 PPI network. Each supgraph has different color and pattern

Here we briefly analyze the motifs identified by our method on the hhv-8 PPI network which causes Kaposi’s sarcoma disease. We choose this organism in our case study as it has the smallest PPI network among the organisms in our database (see Table 1). Notice from Fig. 11 c that despite its small size (48 nodes and 82 edges), hhv-8 has four disjoint embeddings of a very large motif with 10 nodes, covering a significant fraction of its PPI network. This begs the question whether there is a fundamental recurring function that hhv-8 serves and is covered through evolutionary process with high redundancy. Figure 14 presents the structure of those four embeddings. Each row of Table 4 lists the Uniprot ids of the ten proteins that contribute to each of those embeddings. Analysis of these proteins in the Gene Ontology database [37] reveals that three of those four embeddings, each contains two proteins one responsible for viral DNA packaging (O40944 and P88919) and one responsible for virion assembly (P88954). Without either process, no infectious progeny virus could be formed [38]. Several studies use these two processes as targets to identify effective inhibitors. The existence of these two process in each of the three instances reflects the functional importance of the motif topology found. These results suggest that our algorithm can find significant and valuable motifs which can be use to detect key functions governed by the network processes.

**Table 4:** Each row lists the Uniprot IDs of the proteins in an embedding of the most abundant motif of size 10 found by our method in hhv-8 PPI network

O40944	P88947	P88935	P88951	P88960	P88940	P90489	P88918	P90495	P88902
O40910	O40944	P88947	P88929	P88920	P88925	P88927	P90486	P88918	P88954
P88918	P88919	P88929	P88948	P88920	P88950	O36551	P88942	Q98141	P88954
O40944	Q98141	P88920	P88951	P88954	P88947	P88948	P88958	P88939	P88944

## Conclusions

In this paper, we developed a scalable method to solve the motif identification problem given an input graph, desired motif size *μ*, and minimum frequency of desired motif *α*. We proposed a set of small patterns, we call *basic building patterns* each containing two or three edges. We proved that any motif with four or more edges can be constructed as a join of these patterns. Our method first locates instances of the basic building patterns. It then iteratively grows known motifs at that iteration by joining them with the instances of these patterns. We developed efficient mechanisms to avoid a significant fraction of the costly isomorphism tests. We also introduced a new and efficient strategy for solve the MIS extraction problem. We analyzed the time complexity of our method based on the number of nodes and edges in the target network and the number of frequent motifs at each iteration. Our experiments on PPI networks from MINT comprehensively demonstrated that our method is significantly faster and more accurate than the existing methods. Furthermore, we observed using synthetic networks that the running time of our algorithm is reasonable with growing the size of the target network and network density. We also showed using PPI networks that the increase in the running time of our algorithm is dramatically less than that of the competing methods as the motif size grows. We evaluated the statistical significant of the most abundant motif of PPI networks resulting from our algorithm.

## Additional file

Additional file 1 Appendix 1. This appendix shows the algebraic derivation of number of embeddings for each three of the four basic building blocks (see Section 1). In addition, the appendix lists further experimental analysis. Appendix file is attached as PDF file. (ZIP 151 kb)

## Abbreviations

FBF: Flexible pattern finder
MIS: Maximum independent set
PPI: Protein-protein interaction
