# Supplementary material for: Identification of large disjoint motifs in biological networks
Source: BMC Bioinformatics. 2016 Oct 6;17:408. doi: 10.1186/s12859-016-1271-7 (PMC5053092; doi:10.1186/s12859-016-1271-7)
Supplement: Additional file 1 — Appendix 1. This appendix shows the algebraic derivation of number of embeddings for each three of the four basic building blocks (see Section 1). In addition, the appendix lists further experimental analysis. Appendix file is attached as PDF file. (ZIP 151 kb) [file 12859_2016_1271_MOESM1_ESM.zip › Appendix 1.pdf]

Appendix

Finding maximum independent set: Going from F1 to F2

Recall that we discussed how we compute the $F2$ frequency for one pattern, M1 (Figure 1(a)), of the basic building pattern by calculating number of overlaps algebraically instead of constructing the overlap graph. Here we explain how we calculate number of overlaps for the rest of the four basic building patterns. For M2 (Figure 1(b)), we say that two subgraphs overlap if they share only one of the three edges in this pattern accept if they are identical (i.e. both are same subgraph). Lets us denote a vector with length equal to number of edges in the underlying network with VE. Each edge index in VE represents number of subgraphs isomorphic to M2 and this edge is one of their three edges. Let us denote a subgraph isomorphic to M2 with $S_1 = \{e_1, e_2, e_3\}$ where $\{e_1, e_2, e_3\}$ are the indexes of the three edges of S_1. The number of overlapped embeddings of M2 with S_1 is the summation of number of subgraphs that share each of those three edges accept for the three edges within S_1 itself. Thus, the total number of overlaps for the embedding $\{e_1, e_2, e_3\}$ is

$$VE(e_1) + VE(e_2) + VE(e_3) - 3$$

For M3 (Figure 1(c)), we follow a similar calculation as we do for M1. Figure 2(b) shows a hypothetical subgraph $S_2 = \{(a, c),\ (f, c),\ (b, c)\}$ which is an embedding of M3. Another embedding overlaps with this one only if it has one or two from the set of edges $\{(a, c), (b, c),\ (f, c)\}$. Let us first consider the case that there is only one edge that overlaps. Any two edges connected to the left node a, excluding the edge (a, c) itself, forms one overlap with S_2 by sharing the edge (a, c). Thus, we calculate number of overlaps from node a by selecting two edges from its degree, excluding (a, c), which is equal to $\binom{d(a)-1}{2}$. Similarly, node f and node b produce $\binom{d(f)-1}{2}$ and $\binom{d(b)-1}{2}$ overlaps respectively. Likewise, any two edges connected to the middle node c will produce three overlaps sharing either (a, c), or (f, c), or (b, c). Thus, after excluding the three edges of S_2 itself from c' s degree before selecting the two edges, c produces $3 \times \binom{d(c)-3}{2}$ overlaps. Now let us consider the case with two edges overlap. This case occurs only with the middle node c. Any one edge connected to c, produce three overlapping embeddings, one with subgraph that has edge the (a, c), one with subgraph that has edge the (f, c) and the other with subgraph that has the edge (b, c) excluding those three edges themselves. Then node c yields 3 × ($d(c)$ - 3) overlaps according to the second case. Thus, the total number of overlaps for the S_2 is

$$\binom{d(a)-1}{2} + \binom{d(b)-1}{2} + \binom{d(f)-1}{2} + 3 \times \binom{d(c)-3}{2}$$
$$+ 3 \times (d(c) - 3)$$
$$= \binom{d(a)-1}{2} + \binom{d(b)-1}{2} + \binom{d(f)-1}{2} + 3 \times \binom{d(c)-2}{2}$$

For motif M4 (Figure 1(d)), the derivation is more complex than previous patterns. Figure 3(b) shows a hypothetical subgraph $S_3 = \{(x, y),\ (y, z),\ (z, w)\}$ that matches M4. Here we explain three cases of overlaps and how we calculate number of overlaps according to each case. Case 1 (Figure 3(b)) states that only one edge overlaps and this edge has to be one of the two tail edges of the overlap graph. Here, we consider the edge (x, y) as the shared edge with S_3. Each edge connected to a neighbor of x forms embedding of M4 which is overlapped with S_3. Let us denote the set of neighbors of a node with the function $N()$ (i.e. $N(x)$ is the set of neighbors of a node x). In addition, let us denote the sum of neighbors' degrees of a node with function $dn()$ (e.g. $dn(x)$ is sum of degrees of neighbors of node x). We consider excluding y from x's neighbors as one of the nodes in the subgraph S_3 itself. Moreover, while counting the the degree of x's neighbors, we consider excluding edges that are connected to x itself. Thus, the number of overlaps

from neighbors of x is $\sum_{j\in N(x)|j\neq y}(d(j)-1)$ or simply $dn(x)-d(x)-d(y)+1$. Similarly, the overlaps from neighbors of y, z, and w are $2\times(dn(y)-d(y)-d(x)-d(z)+2)$, $2\times(dn(z)-d(z)-d(w)-d(y)+2)$, and $dn(w)-d(w)-d(z)+1$ respectively.

In case 2 (Figure 3(c)), we also consider one edge overlap. However, in this case, this edge is the middle edge of the overlapped subgraph. Any edge connected to y (accept (x,y)) with an edge connected to x (accept (x,y)) forms with the edge (x,y) an overlapped subgrahs with S_3 that is isomorphic to M4. We exclude the edge connects z and y as this will form an overlapped subgraph which is calculated in case 1. Thus, the number of overlaps from case2 according to edge (x,y) is $(d(x)-1)\times(d(y)-2)$. Similarly, when considering other edges (y,z) and (w,z), the number of overlaps are $(d(y)-2)\times(d(z)-2)$ and $(d(w)-1)\times(d(z)-2)$ respectively.

Case 3 considers two edge overlap between two embeddings. Figure 3(d) show a hypothetical subgraph that satisfies this case where the two edge overlapped are (x,y) and (y,z). In this case, each edge connected to either x (accept for the one connects to y) or z (accept for the two connects z with w and y) forms an overlapped subgraph with S_3. Thus, the number of overlaps considering those two edges is $(d(x)-1)$ + $(d(z)-2)$. Similarly, the number of overlaps considering overlaps from two edges (y,z) and (z,w) is $(d(y)-2)$ + $(d(w)-1)$.

Finally, let us consider the special cases to exclude from our calculations. First special case is where neighbors of one node in S_3 is already one of the other nodes in S_3. More specifically, there is an edge connects x and z or x and w or y and w. In those cases we should consider subtracting those edges from the total number overlaps. We subtract 5 overlaps in each of those cases where there is edge connects (x and z) or (y and w) and we subtract only one when the case that x and w are connected. Let us denote the number we should subtract in each of those three cases with function $f()$ (e.g. $f(x,z)=5$ if x and z are connected and $f(x,z)=0$ otherwise). Another special case that we should consider is when a neighbor of one node in S_3 is the same neighbor of another node in S_3 (see Figure 3(e)). In this case, we must subtract 3 $\times$ the number of motifs M2 (Figure 1(b)) that share any of the three edge in M4. In our example, let us denote counts of motif M2 that share any other the three edges (x,y), (y,z), and (z,w) with $countM2(x,y)$, $countM2(y,z)$, and $countM2(z,w)$ respectively. So the total number of overlaps of S_3 is

$$
\begin{aligned}
&dn(x)-d(x)-d(y)+1+2\times(dn(y)-d(y)-d(x)-d(z)+2)\\
&+2\times(dn(z)-d(z)-d(w)-d(y)+2)+dn(w)-d(w)-d(z)+1\\
&+(d(x)-1)\times(d(y)-2)+(d(y)-2)\times(d(z)-2)\\
&+(d(w)-1)\times(d(z)-2)\\
&+(d(x)-1)+(d(z)-2)+(d(y)-2)+(d(w)-1)\\
&-f(x,z)-f(x,w)-f(y,w)\\
&-3\times countM2(x,y)-3\times countM2(y,z)-3\times\\
&countM2(z,w)\\
&=dn(x)+2\times dn(z)+2\times dn(y)+dn(w)\\
&+d(x)d(y)+d(y)d(z)+d(w)d(z)\\
&-4\times d(x)-7\times d(y)-7\times d(w)-4\times d(z)+12\\
&-f(x,z)-f(x,w)-f(y,w)\\
&-3\times countM2(x,y)-3\times countM2(y,z)-3\times\\
&countM2(z,w)
\end{aligned}
$$

Extended Results

Here we extend our results to show how our method perform against SUBDUE if we increase the cutoff frequency, α to 2. We consider same results from experiment in Section 3.2.1. However, we filter out all motifs that exist only once in the underlying network. We perform this process for both our method and SUBDUE. Figures A4, A5, and A6 present

the results. We donot show the frequency of the most abundant motif since the results will not change with changing the cutoff frequency, α.

The results demonstrate that our method significantly outperform SUBDUE in terms of both the number of unique motif topologies found and the average frequency per motif in the target graph especially for large motif sizes (i.e ¿ 5 nodes).

Figures

Figure A1: The four basic patterns used by our algorithm.

Figure A2: (a) One of the four basic building patterns. (b) A hypothetical graph that contains subgraphs isomorphic to the pattern M3 in (a).

Figure A3: (a) One of the four basic building patterns. We call the left and right edges (ends of the pattern) tail edges, and cases for calculating overlaps (b) Case1: one edge overlap from one of the two tails of the overlap graph. (c) Case2: one edge overlap, the middle edge of the overlap graph. (d) Case3: Two edge overlaps. (e) Special case to be considered.

Figure A4: The accuracy of our method (MD) and SUBDUE in terms of three measures (a) the number of unique motif topologies found, and (b) the average frequency per motif in the target graph. The results are for the motif size $\mu = 5$ on the real dataset (see Table 1).

Figure A5: The accuracy of our method (MD) and SUBDUE in terms of three measures (a) the number of unique motif topologies found, and (b) the average frequency per motif in the target graph. The results are for the motif size $\mu = 10$ on the real dataset (see Table 1).

Figure A6: The accuracy of our method (MD) and SUBDUE in terms of three measures (a) the number of unique motif topologies found, and (b) the average frequency per motif in the target graph. The results are for the motif size $\mu = 15$ on the real dataset (see Table 1).
