# Supplementary material for: Identification of large disjoint motifs in biological networks
Source: BMC Bioinformatics. 2016 Oct 6;17:408. doi: 10.1186/s12859-016-1271-7 (PMC5053092; doi:10.1186/s12859-016-1271-7)
Supplement: Additional file 1 — Appendix 1. This appendix shows the algebraic derivation of number of embeddings for each three of the four basic building blocks (see Section 1). In addition, the appendix lists further experimental analysis. Appendix file is attached as PDF file. (ZIP 151 kb) [file A2.pdf › A2.pdf]

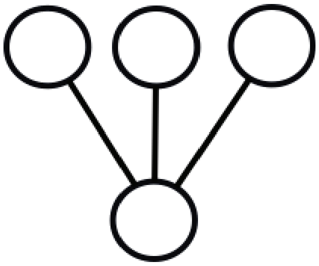

(a) M3

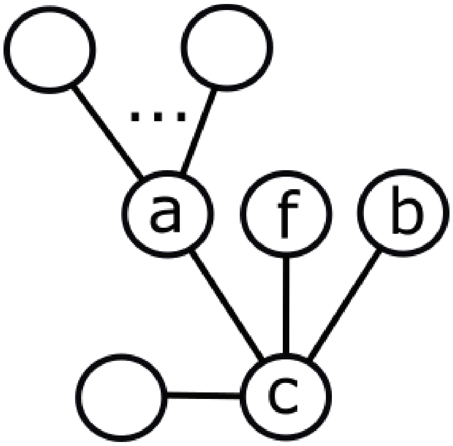

(b)
